# Initial Experience of Minimally Invasive Concomitant Aortic and Mitral Valve Replacement/Repair at a Tertiary Care Cardiac Centre of a Developing Country

**DOI:** 10.7759/cureus.5707

**Published:** 2019-09-20

**Authors:** Kashif Zia, Ali R Mangi, Hafeezullah Bughio, Khuzaima Tariq, Pervaiz A Chaudry, Musa Karim

**Affiliations:** 1 Cardiac Surgery, National Institute of Cardiovascular Diseases, Karachi, PAK; 2 Cardiac Surgery, National Institute of Cardiovascular Disease, Karachi, PAK; 3 Miscellaneous, National Institute of Cardiovascular Diseases, Karachi, PAK

**Keywords:** double valve replacement, developing country, direct vision minimal invasive

## Abstract

Introduction

Minimally invasive double valve replacement (DVR) surgery through a small transverse anterior thoracotomy is an alternate technique to sternotomy for concomitant aortic and mitral valve (AVR, MVR) surgery. The aim of this study was to evaluate the in-hospital and early outcomes of direct vision minimal invasive double valve surgery (DVMI-DVR) at a tertiary care cardiac center of a developing country.

Methods

This study was conducted at the National Institute of Cardiovascular Diseases Karachi, Pakistan from January 2018 to September 2018. Nineteen consecutive patients undergoing DVMI-DVR for aortic and mitral disease without any prior cardiac surgery were included in this study. For all procedures, access was obtained through small transverse anterior thoracotomy incision with wedge resection (Chaudhry’s Wedge) of sternum opposite to the third and fourth costosternal joints. Patients were observed during their hospital stay and the following variables were observed the length of hospital stay (LOHS), ventilator support, intensive care unit (ICU) stay, pain score, and mortality. The pain score was assessed using the visual analog scale (VAS).

Results

The male/female ratio was 11:8 with a mean age of 35 ± 12 years with mean EuroSCORE of 6.6 ± 3.5%. The mean total bypass time was 129.8 ± 23.83 min (range: 98-181 minutes). The mean mechanical ventilation time was 3.16 ± 1.12 hours (range: 2-6 hours). The mean intensive care unit (ICU) stay was 41.84 ± 8.36 hours. The mean post-operative LOHS was 5.63 ± 1.12 days (range: 4-8 days). We had zero frequency of wound infection and surgical mortality. The mean pain score was 4.32 (on a predefined pain scale of one to nine with a high value indicating severe pain).

Conclusion

Minimally invasive DVR surgery is a safe and reproducible technique with comparable outcomes such as postoperative pain score (4.32 ± 2.05), ventilation time (3.16 ± 1.12 hours), ICU stay (41.84 ± 8.36 hours), and hospital stay (5.63 ± 1.12 days). In terms of mortality, operative times, ICU stay, and hospital stay, the minimally invasive DVR is at least comparable to those achieved with median sternotomy. Further prospective randomized studies are needed to validate our findings.

## Introduction

Rheumatic heart disease (RHD) and rheumatic fever are endemic in low- and middle-income developing countries like Pakistan. A study estimated an incidence rate of 5.7% per 1000 individuals in Pakistan [1]. It has a rapid progression leading to death and disability at a young age and remains a leading cause of premature mortality and morbidity in Pakistan [2-3]. Rheumatic fever has been almost completely eradicated in high-income countries, which results in less prevalent mitral-aortic valve diseases [4]. 

Combined surgery for aortic and mitral valve disease was first introduced in the early 1960s; however, over the preceding decade, some reluctance remained in referring a patient for double valve surgery due to high operative mortality [5]. In-hospital mortality in double valve surgery ranges from 5% to 15% and the survival rate at 10 years was reported to be 50% to 70% [5]. The double valve replacement/repair (DVR) is the standard surgical management option for patients requiring surgical management of aortic and mitral valve disease [6-8].

Physicians and health sciences are continuously working to develop newer techniques and methods to improve surgical outcomes and cosmesis. Minimally invasive valve surgery was first performed in the year 1996 by Navia et al. and later followed by Cohn et al. [9-10]. It is associated with a shorter length of hospital and intensive care unit (ICU) stay with enhanced recovery, lesser post-operative pain, and lesser blood loss during the procedure [11-16]. Minimally invasive DVR surgery through a small transverse anterior thoracotomy is an alternate technique to sternotomy for concomitant aortic and mitral valve (AVR, MVR) surgery that can reduce surgical stress and length of hospital stay [17-18].

Endoscopy and robot-assisted surgery are being practiced in developed countries but it is technically very difficult, time-consuming, costly and not reproducible by all surgeons. As median sternotomy is the preferred approach for DVR, therefore, we aimed to assess our initial experience of direct vision minimal invasive DVR (DVMI-DVR) in our setting and to evaluate the in-hospital and early outcomes of direct vision minimal invasive double valve surgery at a tertiary care cardiac center of a developing country.

## Materials and methods

This observational study was conducted at the National Institute of Cardiovascular Diseases Karachi, Pakistan from January 2018 to September 2018. A total of 19 consecutive patients between 18 and 55 years of age, undergoing DVMI-DVR for aortic and mitral disease without any prior cardiac surgery, were included in this study.

**Figure 1:**
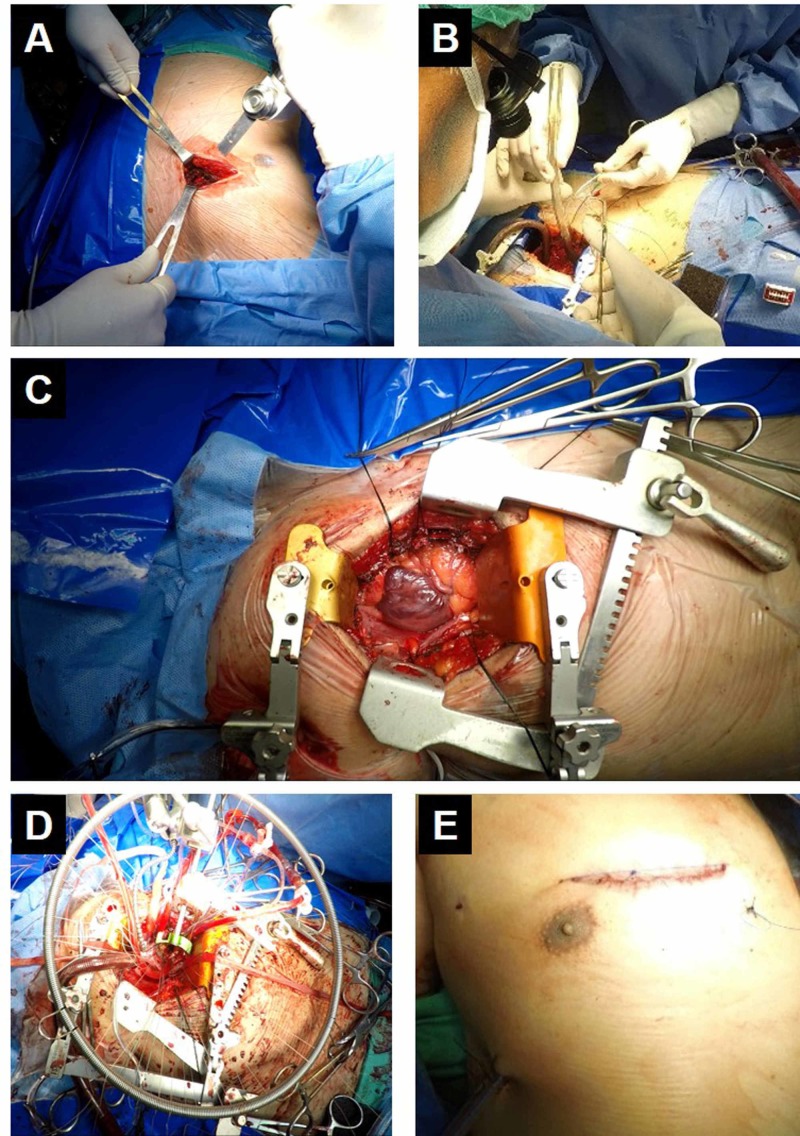
Direct vision minimal invasive double valve replacement A) Oscillating saw being used for Chaudhry’s wedge; B) surgeon performing central cannulation; C) operative exposure with retractors and stay sutures in place; D) mitral valve exposure with metal arm retractor; E) final wound size

For all procedures, access was through small transverse anterior thoracotomy incision with wedge resection (Chaudhry’s Wedge) of sternum opposite to third and fourth costo-sternal joints. Central cannulation strategy was applied in all cases. Femoral artery cannulation was performed only in one case that had difficult aortic access while we were able to clamp the aorta. Superior and Inferior vena cavae cannulation were central with snares in all cases. After the completion of cannulation, cardiopulmonary bypass was established. Systemic hypothermia was 28 ^o^C. The Vitalitec Cygnet® Flexible Aortic Clamp was used in antegrade (induction) and retroplegia (intermittent maintenance) cardioplegia. Modified St. Thomas’ Hospital solution was repeated every 10 to 15 minutes. Root and right superior pulmonary vein (RSPV) were used for venting. After plegic arrest, we entered the LA through transeptal approach after retracting stitches for right atrial appendage and septum. Metal arm retractor was applied for mitral valve exposure while retraction sutures were used for the aortic valve exposure, as visualized in Figure 1.

All cases had both valves replaced except for one case that had a pliable native mitral valve and was repaired with ring annuloplasty. Pre- and post-procedure echocardiography were performed for the assessment of cardiac function and complications. Patients were observed during the hospital stay and were followed to observe the length of hospital stay (LOHS), ventilator support, pain score, and mortality. Pain score was assessed at third postoperative day using visual analog scale (VAS) with pain severity ranging from one to nine and recorded on a predefined proforma for all patients. Data were entered and analyzed using IBM SPSS Statistics for Windows, Version 21.0. (IBM Corp., Armonk, NY, US).

## Results

The male/female ratio was 11:8 with a mean age of 35 ± 12 years with the mean EuroSCORE being 6.6 ± 3.5%. The mean total bypass time was 129.8 ± 23.83 minutes (range: 98-181 minutes). Pre-operative clinical and echocardiographic characteristics are presented in Table 1.

**Table 1:** Pre-operative clinical and echocardiographic profile MS, mitral stenosis; AS, aortic stenosis; MR, mitral regurgitation; AR, aortic regurgitation; TR, tricuspid regurgitation

Characteristics	Total
	n = 19
Age (years)	35 ± 12 years (17–65)
Gender	
Male	11 (57.9%)
Female	8 (42.1%)
New York Heart Association Class (NYHA)	
I	0 (0%)
II	3 (15.8%)
III	7 (36.8%)
IV	9 (17.4%)
Left ventricular ejection fraction (LVEF %)	44.47 ± 10.79 (25-60)
Disease status in patients	
Severe MS with severe MS	8 (42.1%)
Severe MS with Severe AR	5 (26.3%)
Severe MR with Severe AR	4 (21.1%)
Severe MR, AR, and TR	2 (10.5%)

The mean mechanical ventilation time was 3.16 ± 1.12 hours (range: 2-6 hours). The mean post-operative LOHS was 5.63 ± 1.12 days (range: 4-8 days). We had zero frequency of wound infection and surgical mortality. The mean pain score was 4.32 ± 2.05 (on a predefined pain scale of one to nine with a high value indicating severe pain). Post-operative outcomes are presented in Table 2.

**Table 2:** Post-operative outcomes ICU, intensive care unit

Post-operative outcomes	Total	Male	Female
	n = 19	n = 11	n = 8
Ventilation time (hours)	3.16 ± 1.11	3.00 ± 1.00	3.38 ± 1.30
Length of ICU stay (hours)	41.84 ± 8.36	43.91 ± 9.06	39.00 ± 6.82
Hospital length of stay (days)	5.63 ± 1.11	6.00 ± 1.00	5.13 ± 1.12
Pain score (range: 1 to 9)	4.32 ± 2.05	4.82 ± 2.5	3.63 ± 0.7
In-hospital mortality	0 (0%)	0 (0%)	0 (0%)

## Discussion

During the mid-1990s, efforts to avoid a midline sternotomy led to the development of alternate ways of exposing the heart valves. A parasternal approach was initially advocated by the Cleveland Clinic group but then shifted to an upper midline partial sternotomy and was reported to have similar results as a standard sternotomy [19]. Other partial sternotomy incisions, such as the subxiphoid approach, have also been proposed which consists of a transverse skin incision overlying the xiphoid process with an inverted J-type mini-sternotomy [20]. Advantages of these approaches include central cannulation for cardiopulmonary bypass (CPB) along with good valve exposure [21]. However, the need for the sternal division is not obviated and incisions are less aesthetically pleasing to patients when compared with the right mini-thoracotomy incision [22-23].

Currently, most centers favor the right lateral mini-thoracotomy approach for minimally invasive mitral valve operations. CPB is instituted through the cannulation of femoral vessels [24]. Indications are the same for conventional surgery through a median sternotomy and minimally invasive valve surgery. However, the decision to opt for the minimally invasive approach is greatly influenced by the patient-related factors. For example, the presence of elevated atherosclerotic plaques >2 mm in height in the descending thoracic aorta or arch may increase the risk of retrograde cerebral and other systemic embolization and constitutes a contraindication to femoral artery-perfused minimally invasive valve surgery [23].

In this setting, central cannulation is preferable. Among the other relative contraindications, previous breast reconstruction or implant, a previous right thoracotomy with dense pleural adhesions, presence of significant obesity, and a severe chest deformity such as severe scoliosis or a pectus excavatum are worth mentioning [23].

Although a small, randomized clinical trial demonstrated the feasibility of minimally invasive mitral valve surgery, no large, well-powered clinical trial has compared this approach to standard sternotomy with respect to clinical outcomes, the durability of repair, and markers of patient satisfaction [25-26]. However, considering the growing global interest in the technique and an increasing patient recognition of the availability of minimally invasive approaches, such a trial may be difficult to perform. Therefore, available data on minimally invasive mitral valve surgery rely mainly on prospective observational single-center experiences. Adding to the existing pool of knowledge, we aimed to evaluate the in-hospital and early outcomes of direct vision minimal invasive double valve surgery.

One of the primary concern about the minimal invasive approach is whether it is a good trade-off of minimal surgical incision and safety of the established conventional approach [11]. In this study, we observed that ventilation time, ICU stay, hospital stay, and post-operative pain score were comparable to the conventional sternotomy approach with no in-hospital mortality. The safety of the minimally invasive approach was reported by Sharony et al. with no deep wound infections, shorter hospital stays, lesser blood products requirement, and higher five-year survival as compared to median sternotomy [27]. Mihaljevic et al. reported equal or better outcomes of minimally invasive valve surgery as compared to full sternotomy [11]. Similarly, according to Pfannmüller et al., minimally invasive right thoracotomy is a safe option for the tricuspid valve repair/ replacement [28]. Modi et al. conducted a systematic review and meta-analysis of 11 studies comparing safety or outcomes of minimal invasive approach against the conventional approaches and concluded the durability and safety of the minimal invasive approach [16]. Another systematic review by Lucà et al. reported various benefits of minimally invasive mitral valve surgery including improved postoperative respiratory function, decreased postoperative pain, and reduced surgical trauma with comparable long-term efficacy [29].

## Conclusions

Minimally invasive DVR surgery is a safe and reproducible technique with comparable outcomes such as postoperative pain score (4.32 ± 2.05), ventilation time (3.16 ± 1.12 hours), ICU stay (41.84 ± 8.36 hours), and hospital stay (5.63 ± 1.12 days). In terms of mortality, operative times, ICU stay, and hospital stay, the minimally invasive DVR is at least comparable to those achieved with median sternotomy. Further prospective randomized studies are needed to validate our findings.
